# Poor medication adherence and risk of relapse associated with continued cannabis use in patients with first-episode psychosis: a prospective analysis

**DOI:** 10.1016/S2215-0366(17)30233-X

**Published:** 2017-08

**Authors:** Tabea Schoeler, Natalia Petros, Marta Di Forti, Ewa Klamerus, Enrico Foglia, Robin Murray, Sagnik Bhattacharyya

**Affiliations:** a Department of Psychosis Studies, Institute of Psychiatry, Psychology & Neuroscience, King's College London, London, UK

## Abstract

**Background:**

Cannabis use following the onset of first-episode psychosis has been linked to both increased risk of relapse and non-adherence with antipsychotic medication. Whether poor outcome associated with cannabis use is mediated through an adverse effect of cannabis on medication adherence is unclear.

**Methods:**

In a prospective analysis of data acquired from four different adult inpatient and outpatient units of the South London and Maudsley Mental Health National Health Service Foundation Trust in London, UK, 245 patients were followed up for 2 years from the onset of first-episode psychosis. Cannabis use after onset of psychosis was assessed by self-reports in face-to-face follow-up interviews. Relapse data were collected from clinical notes using the WHO Life Chart Schedule. This measure was also used to assess medication adherence on the basis of both face-to-face interviews and clinical notes. Patients were included if they had a diagnosis of first-episode non-organic or affective psychosis according to ICD-10 criteria, and were aged between 18 and 65 years when referred to local psychiatric services. We used structural equation modelling analysis to estimate whether medication adherence partly mediated the effects of continued cannabis use on risk of relapse. The primary outcome variable was relapse, defined as admission to a psychiatric inpatient unit after exacerbation of symptoms within 2 years of first presentation to psychiatric services. Information on cannabis use over the first 2 years after onset of psychosis was investigated as a predictor variable for relapse. Medication adherence was assessed as a mediator variable on the basis of clinical records and self-report data. Study researchers (TS, NP, EK, and EF) rated the adherence.

**Findings:**

397 patients who presented with their first episode of psychosis between April 12, 2002, and July 26, 2013 had a follow-up assessment until September, 2015. Of the 397 patients approached for followed up, 133 refused to take part in this study and 19 could not be included because of missing data. 91 (37%) of 245 patients with first-episode psychosis had a relapse over the 2 years of follow-up. Continued cannabis use predicted poor outcome, including risk of relapse, number of relapses, length of relapse, and care intensity at follow-up. In controlled structural equation modelling analyses, medication adherence partly mediated the effect of continued cannabis use on outcome, including risk of relapse (proportion mediated=26%, β_indirect effects_=0·08, 95% CI 0·004 to 0·16), number of relapses (36%, β_indirect effects_=0·07, 0·003 to 0·14), time until relapse (28%, β_indirect effects_=–0·26, −0·53 to 0·001) and care intensity (20%, β_indirect effects_=0·06, 0·004 to 0·11) but not length of relapse (6%, β_indirect effects_=0·03, −0·03 to 0·09). The adjusted models explained moderate amounts of variance for outcomes defined as risk of relapse (*R*^2^=0·25), number of relapses (*R*^2^=0·21), length of relapse (*R*^2^=0·07), time until relapse (*R*^2^=0·08), and care intensity index (*R*^2^=0·15).

**Interpretation:**

Between 20% and 36% of the adverse effects of continued cannabis use on outcome in psychosis might be mediated through the effects of cannabis use on medication adherence. Interventions directed at medication adherence could partly help mitigate the harm from cannabis use in psychosis.

**Funding:**

This study is funded by the National Institute of Health Research (NIHR) Clinician Scientist award.

## Introduction

Risk of relapse after the first episode of psychosis is high,^1^ constituting a substantial burden for health-care systems around the world,^2^, ^3^ and this relapse affects both individuals and society at large.^4^ In particular, relapse during the first few years after onset of the psychotic episode is an important determinant for long-term clinical and functional outcome.^5^ Hence, prevention of relapse is a crucial treatment target, which in turn underscores the importance of identification of modifiable risk factors that could influence relapse. Although the multifactorial nature of relapse is well known,^6^ two consistently identified modifiable risk factors influencing relapse are continued cannabis use following onset of psychosis^7^, ^8^, ^9^ and medication non-adherence,^10^, ^11^ both of which are unlikely to be the result of confounding or reverse causation.^12^ Despite the fact that the prevalence of post-onset cannabis use^13^ and medication non-adherence^12^ in patients with psychosis is high, understanding of the effects of this remains poor. There is poor understanding about how risk factors such as cannabis use might affect outcome in psychosis. Previous studies^9^, ^14^ have shown that the effect of cannabis use on risk of relapse was reduced when medication adherence was controlled for, suggesting that cannabis use could adversely affect psychosis outcome partly by influencing adherence to antipsychotic medication. This is consistent with independent evidence from a meta-analysis^15^ suggesting a significant effect of continued cannabis use on adherence to antipsychotic medication in patients with psychosis (p<0·0001), which was also confirmed by the five studies^9^, ^12^, ^16^, ^17^, ^18^ that investigated this issue subsequently. However, no study to date has systematically investigated to what extent the association between cannabis use and relapse of psychosis is mediated by non-adherence with prescribed psychotropic medication. By elucidating the mechanistic pathway from cannabis use to psychosis relapse in first episode of psychosis in terms of potential mediational processes, we might be able to help identify alternative targets for intervention that could help mitigate the harm from cannabis use. Hence, in the present study, we aimed to explore whether some of the adverse effects of continued cannabis use on risk of relapse can be explained by its association with medication adherence; whether the association between continued cannabis use and risk of relapse is only partly, but not fully, mediated by medication adherence; and whether mediation effects are also present for other relapse-related outcomes, including number of relapses, length of relapse, time until relapse occurs, and intensity of care (eg, low-intensity outpatient care or high-intensity involuntary admission under section).

**Research in context**

**Evidence before this study**

We searched MEDLINE databases from inception to April 12, 2017, using a combination of search terms for describing diagnosis (psychosis: “psychosis”, “psychot*”, “schizophren*”, “schizoaff*”), exposure (cannabis use: “cannabi*”), and outcome of interest (medication adherence: “adheren*”, “complian*”), which retrieved 2092 articles, of which 20 were selected according to the following three criteria: (1) investigated the relationship between cannabis use and medication adherence; (2) the majority of the sample were taking antipsychotic medication; (3) participants were diagnosed with schizophrenia or any psychotic disorder using standardised criteria. We have previously summarised 15 of these studies (those published before April 27, 2015) as part of a meta-analysis, and these results showed that continued cannabis use increased the risk for non-adherence to antipsychotic medications. Results of the five additional studies that had been published since the original literature search were consistent with the results of our previous meta-analysis and confirmed that cannabis users were less likely to adhere to their prescribed medication than people who did not use cannabis. Of all relevant studies, only one investigated whether the effect of cannabis use on medication adherence mediated its effects on outcome in psychosis. They used data obtained from clinical records to report that poor medication adherence mediated the adverse effect of cannabis use on non-remission at 1 year in patients with psychosis. However, not all patients continue using cannabis following the onset of psychosis and a substantial proportion stop using the drug, a factor that was not accounted for by Colizzi and colleagues. Hence, whether non-adherence to antipsychotics truly mediates the effect of continued cannabis use following the onset of psychosis and the extent of this effect is unclear.

**Added value of this study**

The present study extends current evidence on cannabis use being associated with increased risk of relapse in psychosis by investigating how it might be exerting this effect, focusing particularly on adherence to antipsychotic medication. The study benefits from data obtained in follow-up assessments of a large sample of patients with first-episode psychosis, which allowed a more detailed assessment of cannabis use profiles and pattern of medication adherence and the consideration of potential confounders. We explored consistency of effects by using different outcome measures of relapse, including risk or number of relapses, length of relapse, time until relapse, and severity of relapse.

**Implications of all the available evidence**

Collectively, the results of the present study and previous evidence indicate that relapse of psychosis associated with continued cannabis use might be partly mediated through non-adherence with prescribed medication. Hence, future investigations should test whether interventions aimed at improving medication adherence could partly help mitigate the adverse effects of cannabis use on outcome in psychosis.

## Methods

### Study design and participants

All patients included in this prospective analysis were recruited from four different adult inpatient and outpatient units of the South London and Maudsley (SLAM) Mental Health National Health Service (NHS) Foundation Trust in Lambeth, Southwark, Lewisham, and Croydon as part of a follow-up study aiming to investigate the role of cannabis use within the first 2 years after onset of psychosis. Patients had a clinical diagnosis of first-episode non-organic (non-affective [ICD-10 codes F20–F29] or affective [F30–F33]) psychosis^19^ and were aged between 18 and 65 years when referred to local psychiatric services in south London, UK. We have previously reported on methods for assessment of patients and data acquisition.^9^, ^12^ This study was granted ethical approval by South London & Maudsley NHS Foundation Trust and the Institute of Psychiatry Local Research Ethics Committee. All patients included in the study gave written informed consent.

### Outcomes

We obtained information regarding use of services, including number, duration, and legal status of inpatient admissions and referral to crisis intervention team or standard treatment by a community mental health team from electronic patient records, using the WHO Life Chart Schedule.^20^ Age of onset of psychosis was defined as the age on the date of referral to local psychiatric services for a first episode of psychosis. Our main outcome variable of interest was risk of relapse, which we defined as admission to a psychiatric inpatient unit owing to exacerbation of psychotic symptoms within 2 years following first presentation to psychiatric services. This outcome has been linked to both cannabis use and medication adherence in those with first episode of psychosis.^9^, ^12^ Other relapse-related outcome measures included the number of relapses; the length of relapse; the time to first relapse; the care intensity at follow-up (rating each patient's intensity of service use over the first 2 years following illness onset; appendix).

We assessed cannabis use as a predictor variable using a modified version of the Cannabis Experience Questionnaire (CEQ_mv_),^9^ obtaining data on cannabis use over the first 2 years following onset of psychosis. In line with previous work,^12^ cannabis users were classified on the basis of their pattern of continuation of use after onset, categorising them into different categories (0=not a cannabis user [no use or use only once or twice after onset], 1=intermittent cannabis user [used cannabis more than twice but not every month within the 2-year period], or 2=continued cannabis user [used cannabis every month throughout all of the 24 follow-up months]). The cannabis use variable was coded as ordered categorical.

We assessed medication adherence as a mediator variable within the first 2 years after onset by use of the Life Chart Schedule.^20^ Similar to previous reports,^12^ the variable was classified on the basis of information on prescription and ratings of adherence (3=non-adherence [67–100% of the time non-compliant]; 2=irregular adherence [34–66% of the time non-compliant]; 1=good adherence [0–33% of the time non-compliant], or 0=medication not prescribed within the 2 years following the onset of illness).

Other factors that have previously been reported to be associated with relapse were also included in the model as covariates, including other illicit drug use,^21^, ^22^ ethnic origin,^4^, ^9^ and care intensity at psychosis onset as an index of illness severity when presenting with the first episode.^9^, ^23^ As done in previous studies,^12^ data from the CEQ_mv_ and WHO Life Chart Schedule^20^ were used to derive the following variables. Other drug use was defined as the use of illicit drugs other than cannabis within the first 2 years after onset. This variable was coded as a categorical variable (2=regular use [six times or more]; 1=experimental use [less than six times]; 0=no use). Care intensities at onset and follow-up were computed by rating each patient's intensity of service use at onset or follow-up, respectively.

### Statistical analysis

We created structural equation modelling analyses represented by path diagrams to measure the mediating effect of medication adherence on the association between cannabis use and relapse (appendix). We estimated standardised direct, indirect, and total effects using R and its package Lavaan.^24^ We estimated bias-corrected 95% CIs using 1000 bootstrap samples. The initial simple models estimated path coefficients for continued cannabis use as a predictor for medication adherence, continued cannabis use as a predictor for relapse and relapse-related outcomes, and medication adherence as a predictor for relapse and relapse-related outcomes. As part of the mediation analysis, a direct effect refers to the standardised path coefficient between continued cannabis use and risk of relapse (path C), and an indirect effect to the product of the standardised path coefficient between path A and path B (figure). The total effect of cannabis use on risk of relapse is the sum of direct and indirect effects. Mediation occurred if the indirect effect was significant. Structural equations for each endogenous variable in the pathway model were adjusted for the potential confounding effects of ethnic origin, other illicit drug use, and illness severity at onset as indexed by the level of care intensity at onset. We aimed to further explore an alternative reverse mediation model to compare with the proposed mediation model. In this reverse mediation model for risk of relapse and related outcomes, continued cannabis use was treated as the mediator variable and medication adherence as the independent variable. It is suggested that the predicted mediation model would be more convincing if the reverse model identifies only non-significant indirect paths.^25^

**Figure:**
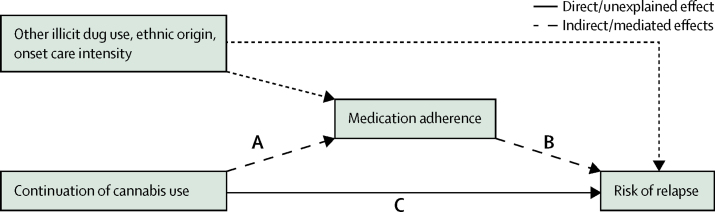
Path model—medication adherence as a mediator of the association between continued cannabis use and relapse Conceptualised pathways between continuation of cannabis use and risk of relapse, with the total effects transmitting both directly (solid line—C), and indirectly (dashed lines—A and B) via medication adherence.

### Role of the funding source

The views expressed are those of the authors and not necessarily those of the NHS, the National Institute of Health Research (NIHR), or the Department of Health. The funders had no role in the study design, data collection, data analysis, data interpretation, or writing of the report. The corresponding author had full access to all the data in the study and had final responsibility for the decision to submit for publication. All authors have approved the final version of the paper.

## Results

397 patients who presented with their first episode of psychosis between April 12, 2002, and July 26, 2013, were approached for follow-up and had an assessment up until September, 2015. Of the 397 patients, 133 refused to take part in this study and 19 could not be included because of missing data. We followed up 245 patients with first-episode psychosis for 2 years from onset. 91 (37%) of 245 patients with first-episode psychosis had a relapse over the 2 years after onset of psychosis (table 1). Most patients reported regular (45%) or irregular (42%) adherence with the prescribed medication, whereas only a small subset of patients (14%) reported non-compliance. Although most patients were classified as not cannabis users following the onset of psychosis (146 [60%], including 98 [40%] who were never a regular user, and 48 [20%] who had been former regular users), the remaining patients were classified either as intermittent cannabis users (36 patients [15%]) or continued cannabis users (63 patients [26%]). Comparing those who relapsed to those who did not relapse revealed that the relapsing patient group was more likely to be classified as continued cannabis users (p=0·0018, 95% CI 0·09–0·43) and as non-adherent (p<0·0001, 0·18–0·58) or irregularly adherent (p=0·0001, 0·13–0·39) with the prescribed medication. With regard to the other demographic and clinical characteristics, the relapsing group was more likely to be of non-white ethnic origin (p=0·015, 0·034 to 0·30) and appeared to have used other illicit drugs more regularly following the onset of first-episode psychosis (p=0·099, −0·04 to 0·41).

**Table 1:** Sample characteristics

		**Total (n=245)**	**Non-relapsing (n=154)**	**Relapsing (n=91)**	p value*	**95% CI**†
Gender, male		147 (60%)	94 (64%)	53 (36%)	0·77	−0·16 to 0·64
Ethnic origin, non-white		161 (66%)	92 (57%)	69 (43%)	0·015	0·034 to 0·30
Age of onset		28·20 (8·11)	28·43 (8·00)	27·81 (8·33)	0·34	−0·89 to 2·53
Onset diagnosis, non-affective		202 (82%)	125 (62%)	77 (38%)	0·61	−0·22 to 0·11
Care intensity at onset‡		..	..	..	0·015	..
	Referral to community team only	40 (16%)	25 (63%)	15 (38%)	..	..
	Required contact with crisis team	18 (<1%)	5 (28%)	13 (72%)	0·030	0·05 to 0·64
	Required hospital admission (non-compulsory)	74 (30%)	50 (68%)	24 (32%)	0·74	−0·26 to 0·15
	Required hospital admission (compulsory)	113 (46%)	74 (66%)	39 (35%)	0·88	−0·22 to 0·16
Other drug use, regular use		26 (11%)	12 (46%)	14 (54%)	0·099	−0·04 to 0·41
Cannabis use‡		..	..	..	0·0034	..
	Never (regular) user	98 (40%)	69 (70%)	29 (30%)	..	..
	Former (regular) user	48 (20%)	35 (73%)	13 (27%)	0·90	−0·20 to 0·17
	Intermittent user	36 (15%)	22 (61%)	14 (39%)	0·42	−0·11 to 0·30
	Continued user	63 (26%)	28 (44%)	35 (56%)	0·0018	0·09 to 0·43
Medication adherence‡		..	..	..	<0·0001	..
	Regular adherence	109 (45%)	86 (79%)	23 (21%)	..	..
	Irregular adherence	102 (42%)	54 (53%)	48 (47%)	0·0001	0·13 to 0·39
	Non-adherence	34 (14%)	14 (41%)	20 (59%)	<0·0001	0·18 to 0·58

Data are n (%) or mean (SD).

* χ^2^ test for independence to compare distributions and Mann-Whitney *U* (two-sided) test to compare means between the groups (non-relapsing *vs* relapsing).

† 95% CI to estimate the difference between two proportions and two means.

‡ χ^2^ test for independence and 95% CI estimate of the difference between two proportions for care intensity at onset (reference group=referral to community team only), cannabis use (reference group=never [regular] user), and medication adherence (reference group=regular adherence).

The simple path models identified the following associations between the variables of interest (table 2): a significant positive association between the level of cannabis use continuation and the risk of relapse, number of relapses, length of relapse, care intensity index at follow-up, and time until a relapse occurred. The proposed mediator, medication adherence, was linked to both the cannabis use variable and the relapse outcome, including risk of relapse, number of relapses, time until a relapse occurred, and care intensity index, but not length of relapse, suggesting that poor adherence was predictive of worse outcome in the 2 years following onset of psychosis.

**Table 2:** Path estimates for cannabis use and medication adherence as predictors for relapse outcome

	**Cannabis (n=245)**				**Medication adherence (n=245)**			
	β (SE)	95%CI	p value	*R*^2^	β (SE)	95%CI	p value	*R*^2^
Risk of relapse	0·35 (0·10)	0·16 to 0·53	0·0003	0·081	0·56 (0·12)	0·33 to 0·80	<0·0001	0·14
Number of relapses	0·28 (0·09)	0·10 to 0·46	0·0019	0·055	0·51 (0·11)	0·28 to 0·73	<0·0001	0·11
Length of relapse	0·53 (0·17)	0·20 to 0·85	0·0016	0·039	0·38 (0·21)	−0·02 to 0·79	0·064	0·01
Time until relapse	−1·11 (0·50)	−2·08 to −0·14	0·024	0·020	−2·33 (0·60)	−3·49 to −1·16)	<0·0001	0·06
Care intensity index	0·32 (0·09)	0·15 to 0·49	0·0002	0·070	0·52 (0·11)	0·31 to 0·74	<0·0001	0·12

Significant association of cannabis use as a predictor for medication non-adherence (β=0·31, SE=0·11, 95% CI 0·093 to 0·53, p=0·0052, *R*^2^=0·046).

Adjusting all models for ethnic origin, other illicit drug use, and intensity of care at onset, the structural equation modelling analyses are reported in table 3. They revealed that the association between continued cannabis use and risk of relapse was mediated (26·4% as the proportion of total effect mediated) by medication adherence (direct effect: β=0·22, 95% CI 0·03–0·42, p=0·027; indirect effect: β=0·08, 0·004–0·16, p=0·040), suggesting a partial but not full mediation by medication adherence of the effect of cannabis use on risk of relapse. For risk of relapse, the model explained 25% of the variance, so 75% is not explained by this model. Explained variance is the extent to which a statistical model accounts for the variation in the dependent variable. The direct effect is the path between continued cannabis use and risk of relapse, and the indirect effect is the product of the path coefficients of the effect of cannabis use and medication adherence and the effect of medication adherence on risk of relapse. A similar effect was seen for care intensity index at follow-up, for which medication adherence mediated 19·7% of the effect of continued cannabis use, again implicating partial mediation based on significant indirect and direct effects. A larger proportion of the effect of continued cannabis use on the number of relapses of psychosis and time until relapse was mediated by medication adherence. No significant mediation effect was present for length of relapse. The adjusted models explained moderate amounts of variance for outcomes defined as risk of relapse (*R*^2^=0·23), number of relapses (*R*^2^=0·21), length of relapse (*R*^2^=0·07), time until relapse (*R*^2^=0·08), and care intensity index (*R*^2^=0·15).

**Table 3:** Mediation of the effect of cannabis use on relapse outcome

		**β (SE)**	**p value**	95% CI*	***R*^2^**
Risk of relapse		..	..	..	0·25
	Indirect effect	0·08 (0·04)	0·040	0·004 to 0·16	..
	Direct effect	0·22 (0·10)	0·027	0·03 to 0·42	..
	Proportion of total effect mediated (%)	26·4%	..	..	..
Number of relapses		..	..	..	0·21
	Indirect effect	0·07 (0·04)	0·040	0·003 to 0·14	..
	Direct effect	0·13 (0·10)	0·20	−0·07 to 0·34	..
	Proportion of total effect mediated (%)	35·5%	..	..	..
Length of relapse		..	..	..	0·07
	Indirect effect	0·03 (0·03)	0·35	−0·03 to 0·09	..
	Direct effect	0·40 (0·18)	0·024	0·05 to 0·75	..
	Proportion of total effect mediated (%)	6·4%	..	..	..
Time until relapse		..	..	..	0·08
	Indirect effect	−0·26 (0·13)	0·051	−0·53 to 0·001	..
	Direct effect	−0·67 (0·52)	0·20	−1·68 to 0·35	..
	Proportion of total effect mediated (%)	28·3%	..	..	..
Care-intensity index		..	..	..	0·15
	Indirect effect	0·06 (0·03)	0·035	0·004 to 0·11	..
	Direct effect	0·24 (0·09)	0·008	0·06 to 0·42	..
	Proportion of total effect mediated (%)	19·7%	..	..	..

Pathways for all endogenous variables were adjusted for other illicit drug use, ethnic origin, and onset care intensity.

* Bias corrected and accelerated 95% CI 1000 bootstrap samples.

Testing of the alternative models with the cannabis use variable as the proposed mediator indicated that continued cannabis use did not mediate the association between medication non-adherence and the different relapse outcomes after controlling for covariates. We tested two different theoretical models, which were a mediation model that tested whether medication adherence mediated the effect of cannabis use on outcome and a reverse arrow model that tested whether cannabis use mediated the effect of medication adherence on outcome.^25^ Studies have used this approach to evaluate which of the proposed mediators (medication adherence or cannabis use) is more valid. In those models, medication adherence had a significant direct effect on risk of relapse (β=0·45, 95% CI 0·20–0·70, p=0·0004), number of relapses (β=0·42, 0·18–0·65, p=0·0005), care intensity index at follow-up (β=0·42, 0·19–0·65, p=0·0003), and time until a relapse occurred (β=–2·00, −3·18 to −0·81, p=0·0010), indicating that cannabis use did not fully confound the effects of medication adherence on outcome. There were no indirect effects for risk of relapse, number of relapses, time until a relapse occurred, and care intensity index at follow-up, which further suggested that cannabis use did not mediate the effects of medication adherence on these relapse outcomes. For length of relapse, there were no indirect or direct effects for medication adherence.

## Discussion

To the best of our knowledge, this is the first study that examines medication adherence as a mediator of the association between continued cannabis use following illness onset and relapse, as indexed by admission to hospital, in patients with first-episode psychosis. The adverse effects of continued cannabis use on risk of relapse were partly but not fully mediated by its association with non-adherence with prescribed antipsychotic medication. More specifically, medication non-adherence mediated the effect of continued cannabis use on risk of relapse (26%), number of relapses (36%), time until a relapse occurred (28%), and care intensity index at follow-up (20%). Medication non-adherence did not mediate the effect of continued cannabis use on length of relapse of psychosis. Our results not only indicate that those patients who continue to use cannabis following onset of their psychotic illness are also more likely to not take medications prescribed for their psychosis but also that this effect can partly explain why patients with first-episode psychosis who continue to use cannabis often suffer from a relapsing form of the illness.^7^, ^26^ We^8^, ^9^ and others^7^ have shown that cannabis use, especially continued use after onset of psychosis, is associated with relapse of psychosis resulting in admission to hospital and that this effect is more likely than not to be a causal association.^12^ This is consistent with other evidence implicating worse outcomes in patients with first-episode psychosis who continued to use cannabis when compared with those who stop using the substance.^27^ Here, we extend this previous work to show that the adverse effect of continued cannabis use on outcome in early psychosis is partly mediated by an effect on adherence with medication treatment. These findings are consistent with studies that identified an association between cannabis use and medication adherence,^8^, ^9^, ^15^, ^28^ as well as between medication adherence and increased risk of relapse of psychosis.^10^, ^11^ We have reported^8^ that failure of treatment with antipsychotic medication as indexed by the number of unique antipsychotic prescriptions could partly mediate the adverse effect of cannabis use on subsequent risk of relapse in first-episode psychosis. Although a change of antipsychotic medication could reflect a clinical judgment of failed treatment, several separate considerations either alone or in combination could lead to such a judgment, including one of treatment resistance, poor tolerability, or non-adherence to a specific antipsychotic. Until now, it has not been known which of these factors might explain how cannabis use could increase the risk of relapse. Results from the present study clearly point toward a mediating influence of poor medication adherence. Whether treatment resistance or poor tolerability also mediate some of the effects of cannabis use on relapse of psychosis is yet to be tested. Furthermore, other factors, such as depressive symptoms^29^ or cognitive function^30^ that were not systematically investigated in this study could also have influenced the association between cannabis use and risk of relapse.

Overall, our results suggest that although efforts should no doubt continue to develop more effective interventions to help patients with psychosis to reduce their cannabis use—eg, similar to those cannabis-focused treatment programmes that are currently under assessment,^31^ another potential approach to mitigating the harm from cannabis use might lie in ensuring better adherence of patients to their prescribed medication. It is worth noting that despite the identified mediation effect, a considerable proportion of the variance in the risk of relapse and related outcomes remains unexplained, varying between 7% and 25% depending on the specific outcome. Future studies including much larger samples are needed to consider other risk factors of interest as well as more complex model pathways to address the issue of unexplained variance in relapse outcome. In this context, it should be pointed out that the identified associations could also be bidirectional. It is worth noting that as the present study was an observational study, temporal ambiguity between the mediator and predictor variable as well as unmeasured confounders could have biased our results. Nevertheless, to partly address this limitation of absence of experimental data, we compared the proposed mediation model with an alternative path model with reversed arrows (ie, by including cannabis use as a mediating factor instead of medication adherence) but the results were not supportive of alternative path models that included cannabis use as a mediator of the associations between medication adherence and relapse outcome (for path estimates see appendix). Although other limitations of this study might relate to the nature of the retrospective assessment of cannabis use and medication adherence, and the inclusion of a selective subset of inner city patients with first-episode psychosis who were at least 18 years old, those issues are unlikely to have affected the results (appendix). We did not consider those who started using cannabis after the onset of psychosis but had no history of premorbid regular use as a separate group, since only three participants belonged to this category. How continued cannabis use might have resulted in poor adherence to medications in psychosis patients is unclear. Although it is possible that increased severity of psychosis,^7^ and consequently, impaired insight or memory^32^ as a result of continued cannabis use might explain poor adherence, this possibility was not investigated in the present study and warrants investigation in the future.

Our results suggest that up to a third of the adverse effect of cannabis use on outcome in first-episode psychosis could be mediated through its effect on medication adherence, suggesting that interventions aimed at improving medication adherence might partly help mitigate the adverse effects of cannabis use on outcome in psychosis.
