# Supplementary material for: Poor medication adherence and risk of relapse associated with continued cannabis use in patients with first-episode psychosis: a prospective analysis
Source: Lancet Psychiatry. 2017 Aug;4(8):627–33. doi: 10.1016/S2215-0366(17)30233-X (PMC5522816; doi:10.1016/S2215-0366(17)30233-X)
Supplement: Supplementary appendix [file mmc1.pdf]

THE LANCET
Psychiatry

Supplementary appendix

This appendix formed part of the original submission and has been peer reviewed. We post it as supplied by the authors.

Supplement to: Schoeler T, Petros N, Di Forti M, et al. Poor medication adherence and risk of relapse associated with continued cannabis use in patients with first-episode psychosis: a prospective analysis. *Lancet Psychiatry* 2017; published online July 10. http://dx.doi.org/10.1016/S2215-0366(17)30233-X.

Online Supplementary Material

Title: Poor medication adherence and risk of relapse associated with continued cannabis use in patients with first episode psychosis: a prospective analysis

Tabea Schoeler, PhD [1]
Natalia Petros, MSc [1]
Marta Di Forti, PhD [1]
Ewa Klamerus, BSc [1]
Enrico Foglia, BSc [1]
Robin Murray, FRS [1]
Sagnik Bhattacharyya, PhD [1]*

*Corresponding author
Department of Psychosis Studies, Institute of Psychiatry, Psychology & Neuroscience, King's College London, De Crespigny Park, London SE5 8AF, UK, Tel +44 207 848 0955, Fax +44 207 848 0976, Email: sagnik.2.bhattacharyya@kcl.ac.

Includes

sMethods
sDiscussion
sReferences

sMethods

Outcome variables: Relapse of psychosis

Information regarding service use, including number, duration and legal status of in-patient admissions, referral to crisis intervention team or standard treatment by a community mental health team was obtained from electronic patient records, using the WHO Life Chart Schedule[1]. For the purpose of this study, age of onset of psychosis was defined as the age on the date of referral to local psychiatric services for a FEP. Our main outcome of interest was risk of relapse, which we defined as admission to a psychiatric inpatient unit owing to exacerbation of psychotic symptoms within two years following first presentation to psychiatric services. This has been proposed as a reliable measure to assess relapse in psychosis[2] and has been linked to both cannabis use and medication adherence in those with FEP[3,4]. Other relapse-related outcome measures (as investigated previously[3]) included: (a) number of relapses [the cumulative number of hospital admissions following the onset of illness over the 2-year period]; (b) length of relapse [the cumulative number of months spent in hospital over the two years following illness onset]; (c) time to first relapse [the consecutive number of survival months without experiencing a relapse]; (d) care intensity at follow up [rating each subject's intensity of service use over the first two years following illness onset (0=Required only community treatment without crisis intervention; 1=Required crisis intervention without hospital admission; 2=Required hospital admission without compulsory admission; 3=Required compulsory hospital admission)].

Statistical analysis

Structural equation modeling (SEM) analyses represented by path diagrams were performed to measure the mediating effect of medication adherence on the relationship between cannabis use and relapse. SEM is considered as a useful tool when examining mediation pathways[5], since it allows the simultaneous evaluation of several equations that is considered to be more powerful and robust than the estimates based on sequential regressions[6] as done using the traditional approach recommended by Baron and Kenny[7]. Standardized direct, indirect and total effects were estimated using R and its package Lavaan[8]. Bias-corrected 95% CIs were estimated using 1000 bootstrap samples. The initial simple models estimated path coefficients for (a) continued cannabis use as a predictor for medication adherence, (b) continued cannabis use as a predictor for relapse and relapse-related outcomes and (c) medication adherence as a predictor for relapse and relapse-related outcomes. As part of the mediation analysis, 'direct effect' refers to the standardized path coefficient between continued cannabis use and risk of relapse (path C), and 'indirect effect' to the product of the standardized path coefficient

between path A and path C. The 'total effect' of cannabis use on risk of relapse is the sum of direct and indirect effects. Mediation occurred if indirect effect was significant. Structural equations for each endogenous variable in the pathway model were adjusted for the potential confounding effects of ethnicity, other illicit drug use and illness severity at onset as indexed by the level of care intensity at onset. Since all models were saturated, it fit the data completely (i.e. 0 df). Since a temporal order between the variables medication adherence, continued cannabis use and relapse is difficult to disentangle in the absence of an experimental design, we aimed to further explore an alternative reverse mediation model to compare with the proposed mediation model. In this reverse mediation model for risk of relapse and related outcomes, continued cannabis use was treated as the mediator variable and medication adherence as the independent variable. It is suggested that the predicted mediation model would be more convincing if the reverse model identifies only non-significant indirect paths[9].

sDiscussion

To partly address the limitation of absence of experimental data, we compared the proposed mediation model to an alternative path model with reversed arrows (i.e. cannabis use as the mediating factor). These results were not supportive of alternative path models that included cannabis use as a mediator of the relationships between medication adherence and risk of relapse ($p_{\text{indirect effect}}$=0·08), number of relapses ($p_{\text{indirect effect}}$=0·13), length of relapse ($p_{\text{indirect effect}}$=0·10), time until relapse ($p_{\text{indirect effect}}$=0·26) or care intensity index at follow up ($p_{\text{indirect effect}}$=0·07). While this may suggest that the proposed model is more valid than the reverse mediation model, this should only be interpreted with caution since a valid test of this matter requires a more tailored experimental design in order to accurately disentangle the precise temporal relations.

While other limitations of this study may relate to the nature of the retrospective assessment of cannabis use and medication adherence, these were validated by screening patients' clinical records. There was also high concordance between cannabis use data collected at onset of illness and at follow-up suggesting minimal risk of under-reporting[3]. Validity of self-report data for measuring adherence and cannabis use have also been demonstrated by comparing with serum concentration of medication[10] and urine drug screen measures[11] respectively.

Arguably, the inclusion of a selective subset of inner city FEP patients, may have affected these results. However, selection bias of less unwell patients is unlikely to have affected the conclusions of our study as engagement/recruitment of patients with more severe psychopathology was unlikely to have been better in those

with comorbid cannabis use[3,4]. It should be noted that we only included FEP patients who were at least 18 years old, which is a commonly applied inclusion criterion in FEP studies. Future studies may also include younger patients to confirm whether the results reported here also apply to FEP patients below the age of 18.

sReferences

1. Susser E, Finnerty M, Mojtabai R, et al. Reliability of the Life Chart Schedule for assessment of the long-term course of schizophrenia. *Schizophr Res* 2000; **42**(1): 67-77.
2. Addington DE, Patten SB, McKenzie E, Addington J. Relationship between relapse and hospitalization in first-episode psychosis. *Psychiatr Serv* 2013; **64**(8): 796-9.
3. Schoeler T, Petros N, Di Forti M, et al. Effects of continuation, frequency and type of cannabis use on relapse in the first two years following onset of psychosis - an observational study. *Lancet Psychiatry* 2016; **3**(10): 947-53.
4. Schoeler T, Petros N, Di Forti M, et al. Examining the association between continued cannabis use and risk of relapse in first episode psychosis: a quasi-experimental investigation within an observational study. *JAMA Psychiatry* 2016; **73**(11): 1173-9.
5. Gunzler D, Chen T, Wu P, Zhang H. Introduction to mediation analysis with structural equation modeling. *Shanghai archives of psychiatry* 2013; **25**(6): 390.
6. Iacobucci D, Saldanha N, Deng X. A meditation on mediation: Evidence that structural equations models perform better than regressions. *Journal of Consumer Psychology* 2007; **17**(2): 139-53.
7. Baron RM, Kenny DA. The moderator–mediator variable distinction in social psychological research: Conceptual, strategic, and statistical considerations. *Journal of personality and social psychology* 1986; **51**(6): 1173.
8. Rosseel Y, Oberski D, Byrnes J. Lavaan: latent variable analysis2011. (accessed.
9. Lam B, Yang Y, Raine A, Lee T. Neural mediator of the schizotypy–antisocial behavior relationship. *Translational psychiatry* 2015; **5**(11): e669.
10. Jónsdóttir H, Opjordsmoen S, Birkenaes AB, et al. Medication adherence in outpatients with severe mental disorders: relation between self-reports and serum level. *Journal of clinical psychopharmacology* 2010; **30**(2): 169-75.
11. Di Forti M, Iyegbe C, Sallis H, et al. Confirmation that the AKT1 (rs2494732) genotype influences the risk of psychosis in cannabis users. *Biol Psychiatry* 2012; **72**(10): 811-6.
